# Exploring the Acute Effects of Sedentary Behaviors on Cardiometabolic Risk in Hispanic Adolescents with Obesity: A Randomized Crossover Study

**DOI:** 10.3390/children12040513

**Published:** 2025-04-16

**Authors:** Erica G. Soltero, Osamah Salim, Yiming Mirabile, Salma M. Musaad

**Affiliations:** USDA/ARS Children’s Nutrition Research Center, Department of Pediatrics, Baylor College of Medicine, 1100 Bates Street, Houston, TX 77030, USAyiming.mirabile@bcm.edu (Y.M.); salma.musaad@bcm.edu (S.M.M.)

**Keywords:** sedentary behaviors, Hispanic health, obesity, screen time, cardiometabolic disease

## Abstract

**Background/Objectives**: Time spent in sedentary pursuits is associated with adverse metabolic profiles. Adolescents spend 65–75% of their day in sedentary pursuits; however, evidence among youth is less conclusive. This study examined the effects of an acute 4 h bout of sedentary behaviors on cardiometabolic outcomes and energy expenditure in Hispanic adolescents (12–16 years old) with obesity (BMI% ≥ 95th). **Methods**: This study used a randomized cross-over design to engage participants (N = 12) in two sedentary conditions, an active condition where youth were engaged in two hours of ‘active’ sitting activities (e.g., reading and puzzles) and two hours of passive movie-watching. Whole-room calorimetry was used to assess energy expenditure. Fasting measures of insulin, adiponectin, leptin, and TNF-alpha were collected, followed by post-prandial measures 30 min, 2 h, and 4 h after a standardized meal. Adiposity was assessed using DXA. **Results**: There was no overall impact of the 4 h sedentary bout on energy expenditure or cardiometabolic risk factors; however, energy expenditure in the active sedentary condition was higher compared to the passive sedentary condition (*p* = 0.0635, ß estimate = 0.1538). Sex and adiposity did not moderate the relationships among sedentary time, cardiometabolic outcomes, and energy expenditure. **Conclusions**: Due to power limitations, these results are exploratory; however, they suggest that different types of sedentary behaviors may be more deleterious than others. More studies are needed to understand the context in which sedentary activities occur and the mechanisms by which sedentary pursuits contribute to the development of cardiometabolic disease.

## 1. Introduction

Over the past two decades, sedentary behaviors have been recognized as independent risk factors for many cardiometabolic diseases, including type 2 diabetes (T2D) [1]. Sedentary behaviors are defined as waking behaviors performed at an energy expenditure of ≤1.5 metabolic equivalent tasks while in a sitting, lying, or reclining position [2,3]. Independent from physical activity or inactivity, it is believed that sedentary behaviors have their own distinct behavioral determinants [4] and even their own distinct physiologic impact on health [5]. This is to say that the mechanistic pathways by which sedentary pursuits may be harmful to one’s health are separate and distinct from the mechanistic pathways by which physical activity may be beneficial for one’s health [5]. The underlying physiologic processes by which sedentary behaviors lead to increased risk for obesity and related metabolic diseases include reduced energy expenditure, increased fat accumulation, increased insulin resistance, and muscle mass loss, all of which lead to metabolic disruption. Adolescence is a critical life stage in which youth experience a precipitous decrease in physical activity and an increase in time spent being sedentary [6]. Adolescents spend an estimated 65–75% of their day in sedentary pursuits [6]. While sedentary behaviors are associated with measures of obesity in larger, nationally representative studies, the relationship between objectively measured sedentary behaviors and cardiometabolic risk factors is less conclusive in youth [7].

Evidence on the physiologic pathways by which sedentary behaviors impact cardiometabolic outcomes has primarily come from immobilization and bed rest studies in adults [7]. These studies have demonstrated that short, acute bouts of sedentariness (3 h of immobilization or 2 days of bed rest) are associated with adverse metabolic outcomes, including insulin resistance, elevated cardiovascular risk factors, and dysregulated energy metabolism [8,9,10]. However, little is known about the physiological changes that accompany prolonged sitting in youth [11]. Additionally, most bed rest or immobilization studies are conducted among otherwise healthy populations [1], leading investigators to believe that youth with obesity, a highly sedentary population, may produce results that are more similar to those observed in adults following an acute bout of sedentariness [12].

Many studies have used screen time as a proxy for sedentary behaviors; however, screen time, which is considered a ‘passive’ sedentary activity, is not a comprehensive measure of all sedentary pursuits as it does not reflect ‘active’ sedentary activities like reading. Passive sedentary pursuits include recreational engagement on a screen-based device like the use of a television, computer, tablet, or smartphone for viewing content, watching movies and videos, browsing the internet, spending time on social media, and playing video games [7,13]. In contrast, active sedentary pursuits are non-screen-based activities that are performed while in a sedentary position (e.g., seated and lying), like studying, reading, or completing a crossword puzzle. It is hypothesized that passive and active sedentary pursuits may have differential effects on health outcomes [13]. Some studies among adults have reported that passive sedentary pursuits are more deleterious for physical and mental health compared to active sedentary pursuits due to differences in exposure to content, postural position, and mental engagement [13]. While recreational screen time has been associated with disease-related outcomes, including higher levels of adiposity, lipids, diabetes indicators, and BMI [7], a knowledge gap still exists regarding active sedentary pursuits and their differential impact on health outcomes.

While daily guidelines exist for physical activity, there are no guidelines or recommendations regarding the type or patterns of sedentary behavior that are optimal for health. To better understand the impact of sedentary behaviors on metabolic health in youth, there is a need for controlled clinical studies in youth [12]. The purpose of this study was to examine the impact of an acute 4 h bout of sedentary behaviors on cardiometabolic outcomes and energy metabolism. The secondary purpose was to examine differences between active and passive sedentary pursuits on cardiometabolic outcomes and energy metabolism. The tertiary purpose of this study was to examine adiposity and sex as mediators. Excess adiposity is one of the underlying mechanisms or mediators by which sedentary behaviors are thought to be more detrimental in individuals with obesity [14]. Lean mass is a significant driver of energy expenditure because it has a higher metabolic rate compared to fat and is commonly tested as a mediator between obesity and energy metabolism [15,16]. However, among youth, the role that lean or fat mass plays in the relationship between sedentary behaviors, cardiometabolic outcomes, and energy metabolism is unknown. During adolescence, youth undergo normal pubertal changes in hormones, metabolism, and body composition, including increased lean mass in boys and increased fat mass in girls [17]. Therefore, in light of these sex-based physiologic differences, we will also examine sex as a mediator of the relationship between the effects of sedentary behaviors on cardiometabolic outcomes and energy metabolism.

## 2. Materials and Methods

### 2.1. Participants

This randomized crossover study included 12 Hispanic adolescents (14–16 years) with obesity (BMI ≥ 95th percentile < 120% of the 95th percentile) based on the Centers for Disease Control and Prevention defined age- and sex-specific BMI percentiles [18,19]. Youth were recruited through the Children’s Nutrition Research Center (CNRC) volunteer database. Youth and parents received an email or phone call inviting them to participate in the study after a brief description of procedures. Participants were screened using the following inclusion criteria: (1) youth participant self-identifies as having Mexican American origin, (2) present with obesity (BMI% ≥ 95th percentile and <120% of the 95th%), and (3) between the ages of 14 and 16 years. We focused exclusively on Mexican American youth as there are metabolic and body composition differences across Latino subgroups. Participants were excluded if they were (1) taking medication(s) or diagnosed with a medical or physical condition that would interfere with cardiometabolic biomarkers or metabolism (e.g., metformin, limb impairment, or GLP-1 agonists); (2) diagnosed with T2D or any endocrine abnormalities (e.g., thyroid disorders or fatty liver disease); or (3) currently enrolled in a formal exercise or weight loss program. This protocol was approved by the institutional review board at Baylor College of Medicine. Parents’ informed consent and youth assent were obtained prior to any study procedures.

### 2.2. Procedures

**Figure 1:**
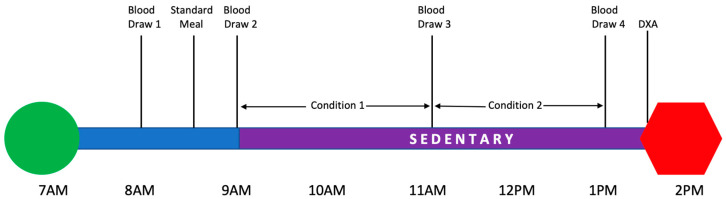
Study design.

The design for this randomized cross-over study is presented in Figure 1. Participants arrived at the metabolic research unit at the CNRC at 7am following an overnight fast. Height and weight were assessed using a wall-mounted stadiometer and electronic scale, respectively. BMI was calculated as body weight in kilograms divided by height in meters squared. Participants completed the pubertal development survey to assess Tanner stage. A standardized meal was administered at 9am. The meal was relative to the estimated daily energy requirements, providing 25% of estimated daily needs with macronutrients consisting of 15% protein, 30% fat, and 55% carbohydrate as analyzed using Nutrition Data System software 2024 (Minneapolis, MN, USA). Throughout the protocol, water was provided ad libitum. The volume of water and food consumed was weighed before and after consumption to compute net energy balance. Youth were studied in the non-fasted state as this is more indicative of a normal day than a fasted state, and four hours was selected as the timeframe for the acute bout of sedentary time as this timeframe is similar to a typical school schedule where students are primarily sitting in class [11]. After the meal was consumed, participants were randomized to begin the acute bout of sedentary behaviors in the active or passive condition. The active sedentary condition consisted of non-screen-based activities performed in the seated position at a desk. In this condition, the participant could select from reading books, puzzles, and coloring book materials. The passive sedentary condition consisted of watching a movie on a television screen. To begin the protocol, youth were escorted into the whole room calorimeter at 10am to begin the bout of 4 h of sedentariness. After two hours in one condition, they crossed over to the alternate condition at 12pm before being removed from the chamber at 2pm. Following the acute bout of sedentary behaviors, body composition was assessed.

### 2.3. Cardiometabolic Outcomes

Fasting insulin, adiponectin, leptin, and TNF-alpha were measured upon arrival at the clinical unit (T0). Additional postprandial measures were taken 30 min after the standardized meal was consumed (T1), after 2 h in the calorimeter (T2), and immediately following the acute bout of 4 h of sedentary time (T3). All blood biomarkers were measured and analyzed using standard procedures in the Metabolic Research Unit at the CNRC (Houston, Texas). These biomarkers were selected as they are strongly associated with energy metabolism and insulin sensitivity, which are central to the pathogenesis of metabolic diseases. Energy metabolism was assessed using a whole-room calorimeter, which has been previously described [20]. In brief, the whole-room calorimeter measures total energy expenditure, and substrate oxidation is determined using oxygen consumption and carbon dioxide. Gas concentrations are determined from the flow rate and the differences in CO_2_ and O_2_ concentrations between entering and exiting air. The metabolic room is sealed, but fresh air is constantly drawn in. Body composition was assessed in the body composition lab using dual X-ray absorptiometry (iDXA; GE Healthcare, Chicago, IL USA) and analyzed using enCore version 16.2 (GE Healthcare).

### 2.4. Statistical Analysis

We conducted a t-test to detect a difference in energy expenditure between the 2 study conditions in a 2 × 2 Cross-Over design. A sample size of 12 individuals provides 88% power to detect a mean difference of 0.3 across conditions, assuming a standard deviation of the paired differences of 0.3, within-individual correlation of 0.5, and a 2-sided 5% significance level. PASS 2021 Power Analysis and Sample Size Software (2021) were used; NCSS, LLC; Kaysville, UT, USA, www.ncss.com/software/pass, accessed on 20 November 2024. Continuous variables were summarized using mean, median, minimum, maximum, and standard deviation. The association between condition (active vs. passive sedentary pursuits) with energy expenditure and cardiometabolic outcomes (insulin, adiponectin, leptin, and TNF-alpha) was examined using separate linear mixed effect models that included condition (movie or desk), sex, group order of the condition (=1 if condition is movie, =2 if condition is desk) and the interaction between condition and group as fixed effects. We specified the compound symmetry covariance structure for the repeated measures and subject nested within group as random effect.

To examine mediators of the association of condition (active vs. passive sedentary pursuits) on energy expenditure, cardiometabolic outcomes were examined using mixed effect models. One model included condition of sex, interaction between condition and sex, group (1 = started in active sedentary condition, 2 = started in passive sedentary condition), fat mass, and lean mass as fixed effects. The other model had the same specification except that we replaced the sex and its interaction with condition with adiposity and its interaction with condition. We specified the unstructured covariance structure for the repeated measures and subject nested within group as random effect. A significance level of 0.05 was used. Analyses were conducted using SAS version 9.4 (SAS Institute, Inc., Cary, NC, USA).

## 3. Results

The purpose of this study was to examine the impact of an acute, 4 h bout of sedentary behaviors on cardiometabolic outcomes and energy metabolism in a sample of Hispanic adolescents (12–16 years) with obesity. Participant demographics are presented in Table 1. We also examined differences between active and passive sedentary pursuits on these primary outcomes and whether adiposity and sex moderated these relationships. In this study, we did not observe any significant associations between the acute 4 h bout of sedentary behaviors on cardiometabolic outcomes or energy expenditure (*p* > 0.05). Statistics from the linear mixed effects are presented in Table 2. It is possible that a four-hour bout of continuous sedentary time was not long enough to produce significant changes in our selected biomarkers. A previously published systematic review of studies involving otherwise healthy adults reported that when acute bouts of sedentary behaviors were less than one day, evidence of significant changes in glycemic and lipid biomarkers was ‘very low-quality’ [1]. The Hispanic Community Health Study/Study of Latinos study, which is the largest known study of health behaviors in Hispanic adults in the U.S., found a joint association between the volume of total time spent in sedentary pursuits and the duration of sedentary bouts with diabetes risk indicators [21]. These findings suggest that in addition to the duration of the sedentary bout, the total sedentary time accumulated throughout one’s day is an important factor, and these two variables together influence disease risk [21].

## 4. Discussion

While we did not find an impact of the 4 h bout of sedentary behaviors on cardiometabolic outcomes and energy metabolism, we did find a significant difference in energy expenditure when youth were engaged in the active sedentary conditions compared to the passive sedentary condition. The difference in energy expenditure between passive vs. active sedentary pursuits may be due to light movements in the upper limbs that can occur during active sedentary activities like coloring or reading [22]. Similarly, it has been hypothesized that these differences are due to differences in posture allocation; for example, one may engage or contract more muscles while sitting upright to color as opposed to reclining to watch television [23]. Consistent with findings from other studies, all participants had MET values below 1.5 while engaged in passive sedentary activities [24,25,26,27,28]. In contrast, all participants recorded MET values above 1.5 when engaged in active but sedentary behaviors, including reading, drawing, and completing brainwork (e.g., crossword puzzles or sudoku). However, studies that showed that some sitting behaviors have a MET threshold greater than 1.5 found that this energy expenditure difference is negligible, with no metabolic advantage [23]. This assertion is upheld in our work as we found no metabolic advantage of the increased energy expenditure during the active vs. passive sedentary condition. However, the implications of having some active sedentary pursuits above the 1.5 MET value threshold in this high-risk population warrants further exploration. Our study demonstrated that the increased energy expendutring during active sedentary pursuits did not lead to significantly different cardiometabolic outcoms as measured within the 4 h protocol. However, future studies with more longitudinal assessment of active vs. sedentary behaviors are needed to understand if differences in energy expenditure impact metabolic health outcomes over time.

The observed increase in energy expenditure found in our active sedentary condition opens the door to further discussion on how to approach physical activity promotion among youth. The American Academy of Pediatrics recommends an average of 60 min of daily physical activity at a moderate to vigorous intensity [29]. Many activity promotion strategies use an elevator approach, meaning they seek to quickly increase activity among participants, encouraging them to quickly transition from being sedentary and physically inactive to meeting the 60 min activity recommendation. However, only 20% of U.S. youth meet this recommendation, and the prevalence is even lower among youth with overweight or obesity [30]. Thus, strategies that use the elevator approach can be daunting, unrealistic, and have shown limited effectiveness [31]. This highlights the growing need to embrace a staircase approach with an emphasis on taking slow steps, making incremental improvements toward completing activity at a moderate to vigorous activity level. This leads us to consider if replacing passive sedentary behaviors with mentally active tasks may be a prudent first step in the staircase toward higher energy expenditure leading to the activity recommendation. Conducting future research in this area can lead to a deeper understanding of the energy and metabolic differences between active and sedentary behaviors, which, in turn, can inform future intervention targets and national guidelines on limiting sedentary behaviors. Currently, there are no specific guidelines on time spent in sedentary pursuits, and clinicians do not have guidance on counseling patients on the duration and type of sedentary behaviors. Additionally, incremental changes, like replacing passive sedentary time with active sedentary time, have been shown to have a positive impact on mental health outcomes like depression and self-esteem [13]. This may be due to reduced time spent engaged in smartphone and social media use, which leads to adverse mental health outcomes among this age group, including some social media use [32].

This study included a small sample size, and therefore, the findings should be interpreted with caution. Additional studies that examine the effects of acute bouts of sedentary behaviors on specific disease outcomes are needed. Future clinical studies should examine the impact of longer bouts of sedentary time on disease outcomes and should explore more varied types of activity, like practicing an instrument, completing homework, or playing a board game, which may lead to further variations in energy expenditure. Future studies should utilize more rigorous measures (e.g., glucose tolerance testing) and measures that can provide more longitudinal data, like continuous glucose monitors, to obtain a better understanding of the metabolic impact of these different sedentary pursuits. Moving forward, the use of traditional (e.g., glucose, insulin, and lipids) and non-traditional disease markers (e.g., markers of inflammation) should also be included to comprehensively explore the health impact of sedentariness [1]. Studies conducted in free-living populations that assess the patterns of sedentary bouts, as well as the total time spent in sedentary behaviors, are also needed to understand how this behavior contributes to disease risk [21]. There is a need for stronger measurement tools that distinguish between passive and active sedentary activities to further examine potential differences among these types of sedentary pursuits. All of this work should strive to identify specific intervention targets for reducing sedentary behaviors among youth [2,21]. As our knowledge of the unique determinants and physiologic pathways of sedentary behaviors increases, more interventions focused on reducing sedentary time will be needed [33,34,35]. The long trajectory of this research should build toward the development of guidelines and recommendations regarding sedentary time among youth and the most effective strategies for breaking up sedentary time [36,37].

## 5. Conclusions

This study aimed to understand the underlying pathophysiology by which sedentary behaviors are associated with cardiometabolic disease risk. However, we found no significant associations between an acute 4 h bout of sedentary time on cardiometabolic outcomes and energy metabolism. Energy expenditure was significantly higher while youth engaged in active sedentary conditions compared to passive sedentary conditions. These findings suggest that more studies are needed to understand how bouts and patterns of sedentary time influence the development of metabolic diseases in high-risk youth. These findings also indicate a need to measure and analyze active and passive sedentary pursuits differently, as they may have a differential impact on metabolic health. Increasing our understanding of sedentary behaviors will inform future strategies aimed at reducing this behavior and will contribute to strengthening national guidelines and recommendations on limiting sedentary behaviors to prevent disease.

## Figures and Tables

**Table 1:** Participant demographics.

Variable	Mean (SD)
Age (years)	15.86 (0.74)
Sex (%)	
Female	66%
Male	34%
BMI (kg/m^2^)	37.66 (7.39)
Parental education	
Elementary education	12.5%
Some high school	37.5%
High school graduate/some college	25%
College graduate	25%
Household income	
≤USD 20,000	25%
USD 20,001–$40,000	37.5%
USD 40,001–$60,000	12.5%
USD 60,001–$80,000	0%
USD 80,001–$100,000	0%
>USD 100,000	25%

**Table 2:** Linear mixed effect examining the association between condition (active vs. passive) with energy expenditure and cardiometabolic outcomes while exploring sex and total fat % as moderators.

Effect	Condition	Group	Sex	Estimate	Standard Error	t Value	*p* Value
**Energy Expenditure**							
Intercept				0.5537	0.2465	2.25	0.0746
Condition	desk			0.01836	0.1305	0.14	0.8936
Sex			Female	−0.01461	0.09649	−0.15	0.8855
Sex × Condition	desk		Female	0.1773	0.2383	0.74	0.4904
Group		1		−0.2433	0.08985	−2.71	0.0424
Fat Mass				0.00000137	0.000002597	0.53	0.6204
Lean Mass				0.000017	0.000003805	4.51	0.0064
Intercept				−0.00021	1.8018	0	0.9999
Condition	desk			0.5221	0.5116	1.02	0.3543
TF%				1.0365	4.3794	0.24	0.8223
TF% × Condition	desk			−1.2077	1.2984	−0.93	0.395
Group		1		−0.2049	0.0989	−2.07	0.0931
Fat Mass				−0.00000146	0.000023	−0.06	0.9515
Lean Mass				0.000021	0.000017	1.2	0.2828
**Insulin**							
Intercept				−96.5986	112.13	−0.86	0.4283
Condition	desk			−26.061	8.9294	−2.92	0.0331
Sex			Female	−25.893	37.418	−0.69	0.5198
Sex × Condition	desk		Female	0.7186	16.303	0.04	0.9665
Group		1		22.4198	32.574	0.69	0.5219
Fat Mass				−0.00021	0.0013	−0.16	0.8757
Lean Mass				0.003688	0.0019	1.96	0.1067
Intercept				−923.66	359.69	−2.57	0.0371
Condition	desk			−168.35	41.8728	−4.02	0.0051
TF%				2418.06	824.15	2.93	0.0219
TF% × Condition	desk			390.45	101.69	3.84	0.0064
Group		1		−70.8918	21.783	−3.25	0.014
Fat Mass				−0.01334	0.004375	−3.05	0.0186
Lean Mass				0.01232	0.00365	3.37	0.0118
**TNF-alpha**							
Intercept				1.7451	0.1061	16.45	<.0001
Condition	desk			0.0097	0.07889	0.12	0.9069
Sex			Female	−0.3624	0.06478	−5.59	0.0025
Sex × Condition	desk		Female	0.02942	0.144	0.2	0.8462
Group		1		−0.2634	0.03191	−8.25	0.0004
Fat Mass				0.000003353	0.000001084	3.09	0.0271
Lean Mass				−0.00002	0.000001588	−10.67	0.0001
Intercept				1.9173	1.4641	1.31	0.2317
Condition	desk			0.1844	0.2898	0.64	0.5448
TF%				−1.1871	3.3699	−0.35	0.735
TF% × Condition	desk			−0.4161	0.7096	−0.59	0.576
Group		1		−0.4313	0.08116	−5.31	0.0011
Fat Mass				0.000007035	0.000019	0.38	0.7179
Lean Mass				−0.00002	0.000016	−1.01	0.3481
**Leptin**							
Intercept				18,880	21,548	0.88	0.421
Condition	desk			3130.54	2409.14	1.3	0.2505
Sex			Female	−8308.28	6914.32	−1.2	0.2833
Sex × Condition	desk		Female	−6767.45	4398.47	−1.54	0.1845
Group		1		−2976.16	7095.18	−0.42	0.6923
Fat Mass				2.4438	0.2441	10.01	0.0002
Lean Mass				−1.1396	0.3576	−3.19	0.0244
Intercept				53,269	131,612	0.4	0.6977
Condition	desk			−12,637	11,550	−1.09	0.3101
TF%				−14,960	301,099	−0.05	0.9618
TF% × Condition	desk			33,987	27,964	1.22	0.2636
Group		1		−12,901	8445.45	−1.53	0.1704
Fat Mass				2.293	1.6156	1.42	0.1988
Lean Mass				−1.4134	1.3474	−1.05	0.329

TF: total fat; Group = 1 if condition is movie.

## Data Availability

The datasets generated and/or analyzed during the current study are not publicly available due to concerns regarding privacy, but select data are available from the corresponding author upon reasonable request.
